# Emotion and affect in mental imagery: do fear and anxiety manipulate mental rotation performance?

**DOI:** 10.3389/fpsyg.2014.00792

**Published:** 2014-07-25

**Authors:** Sandra Kaltner, Petra Jansen

**Affiliations:** Institute for Sport Science, University of RegensburgRegensburg, Germany

**Keywords:** fear, anxiety, mental rotation

## Abstract

Little is known about the effects of fear as a basic emotion on mental rotation (MR) performance. We expected that the emotional arousal evoked by fearful stimuli presented prior to each MR trial would enhance MR performance. Regarding the influence of anxiety, high anxious participants are supposed to show slower responses and higher error rates in this specific visuo-spatial ability. Furthermore, with respect to the embodied cognition viewpoint we wanted to investigate if the influence of fear on MR performance is the same for egocentric and object-based transformations. To investigate this, we presented either negative or neutral images prior to each MR trial. Participants were allocated to the specific emotion in a randomized order. Results show that fear enhances MR performance, expressed by a higher MR speed. Interestingly, this influence is dependent on the type of transformation: it is restricted to egocentric rotations. Both observation of emotional stimuli and egocentric strategies are associated with left hemisphere activation which could explain a stronger influence on this type of transformation during observation. Another possible notion is the conceptual link between visuo-spatial perspective taking and empathy based on the co-activation of parietal areas. Stronger responses in egocentric transformations could result from this specific link. Regarding the influence of anxiety, participants with high scores on the trait-anxiety scale showed poor results in both reaction time and MR speed. Findings of impoverished recruitment of prefrontal attentional control in patients with high scores in trait anxiety could be the explanation for this reduced performance.

## INTRODUCTION

### THE INFLUENCE OF FEAR AND ANXIETY ON COGNITION

According to Ekman (1992) “basic emotions” share nine characteristics which are useful to distinguish emotions from other affective phenomena like moods or emotional traits and attitudes: (1) distinctive universal signals (facial expressions), (2) presence in other primates, (3) distinctive physiology, (4) distinctive universals in antecedent events, (5) coherence among emotional response, (6) quick onset, (7) brief duration, (8) automatic appraisal, (9) unbidden occurrence. According to emotion theorists (Ekman, 1992; Izard, 1992) anxiety as pervasive cognitive affective state has to be differentiated from fear as “basic emotion:” Anxiety represents a higher order cognitive process that depends more on the individual and the situation and is consequently more modifiable than fear. Despite this distinction, they both represent emotional responses to threat (Hofmann et al., 2004).

There is plentiful evidence that fear induced by presenting fearful stimuli affects visual perception in the ventral stream. For instance, Phelps et al. (2006) found that fearful stimuli used as prime produced greater contrast sensitivity compared to neutral faces. Furthermore, regarding early visual areas such as V1 fearful pictures produce higher activation than do neutral ones (Lang et al., 1998). In contrast to this, Schimmack (2005) found that emotional pictures lead to attentional interference resulting in decreased performance while solving math problems or detecting the location of a line. In line with the examination of the influence of fear, there have been several studies to investigate the influence of anxiety on cognitive performance like central executive (Eysenck et al., 2005), inhibition function (Pacheco-Unguetti et al., 2010) or shifting function (Wilson et al., 2009). Anxiety has been linked to poor performance on memory tests such as the digit span (Paulman and Kennelly, 1984) and on more complex cognitive processes such as analogical reasoning (Tohill and Holyoak, 2000).

By analyzing this special relationship, it raises the question whether affect and emotion also have an influence on mental imagery. This study tries to answer this question by investigating the influence of fear and anxiety on a certain visuo-spatial ability, specifically mental rotation (MR).

### THE INFLUENCE OF FEAR AND ANXIETY ON MENTAL ROTATION PERFORMANCE

#### Mental rotation: object-based vs. egocentric transformations

MR involves the process of imagining how a two- or three-dimensional object would look if rotated away from its original upright position (Shepard and Metzler, 1971). In the classic paradigm of Cooper and Shepard (1973), two stimuli are presented simultaneously and the participants have to decide as fast and accurately as possible if the right stimulus, presented under a certain angle of rotation, is the same or a mirror image of the left stimulus, the so-called comparison figure which is presented in upright position. While angular disparities are varied systematically, response times and accuracy rate are assessed as dependent variables.

In MR, there are two different strategies of mental transformations: object-based and egocentric transformations. In object-based transformations, the observer’s position remains fixed and moves the object in relation to the surrounding environment. In egocentric transformation tasks, participants are required to change their own perspective and thus imagine rotating their own body in order to make a decision (Devlin and Wilson, 2010). The use of each strategy depends on the type of judgment that has to be made. In the case of an object-based transformation, participants are asked to perform a same–different judgment for two images presented next to each other. An egocentric transformation can be evoked by the presentation of body stimuli, normally a single human body raising one arm (left or right) and the subsequent decision which arm was raised, thus resulting in a left–right judgment (Steggemann et al., 2011).

#### Specific effects of fear and anxiety on mental rotation performance

Little is known about the effects of fear as basic emotion on visuo-spatial processing in the dorsal stream assessed by MR performance. The corresponding neural system that is activated when a stimulus evokes fear is the amygdala (Phelps, 2006). According to Borst (2013), there are two paths which illustrate the neural processes that underlie the effect of one’s emotional state on MR performance. (1) The amygdala processes the emotional valence of the stimulus and modulates low-level perceptual processing via connections to magnocellular neurons in early visual areas. These areas in turn send efferent projections to higher level visuo-spatial processes such as MR (DeYoe and Van Essen, 1988). The notion of the involvement of the amygdala in early visual processing is supported by the findings of Vuilleumier et al. (2004), who demonstrated that the enhanced responses to fearful stimuli compared to neutral faces were eliminated in patients with amygdala lesions. Furthermore, Borst (2013) confirmed that fear improves MR performance by increasing sensitivity to visual information within the magnocellular pathway. (2) The amygdala is directly connected with parietal areas via structures such as the pulvinar and the superior colliculus (Tamietto and de Gelder, 2010). According to Zacks (2008) parietal areas are considered to be the neural correlate for MR which leads to the assumption that the presentation of fearful stimuli elicits activity in the amygdala which in turn should enhance MR performance due to connections of the amygdala to posterior parietal areas (Borst et al., 2012).

Regarding the influence of anxiety on MR performance, Borst et al. (2012) demonstrated that participants with high anxiety scores mentally rotated Shepard–Metzler three-dimensional objects faster after the presentation of a fearful stimulus than after seeing a neutral face. Since this effect was restricted to the high-anxiety group, the authors concluded that that the increase of the MR speed was a consequence of the emotional arousal evoked by the fearful face which is much higher in the high-anxiety group.

#### Specific effects of fear and anxiety on object-based and egocentric transformations

Despite the fact that the role of emotion and affect has been investigated in the context of MR performance, it is still an open question if the influence of fear and anxiety is the same for egocentric and object-based transformations. The present article addresses this issue with regard to the embodied cognition approach. The key idea of this renewed viewpoint in cognitive neuroscience is that many cognitive processes that were formerly defined as purely “cognitive” are also deeply rooted in body-related experiences with the environment (Wilson, 2002).

There is plentiful evidence that motor processes are involved in both object-based (Wexler et al., 1998; Wohlschläger and Wohlschläger, 1998; Moreau et al., 2012; Pietsch and Jansen, 2012) and egocentric transformations (Steggemann et al., 2011). However, they differ in a crucial point, which is illustrated by the study of Lorey et al. (2009). The authors compared first-person perspective (1PP) to third-person perspective (3PP). 1PP imagery evokes kinesthetic representations and motor simulations. Here, participants are requested to imagine the presented movement kinesthetically as if they were performing it. In contrast, 3PP imagery involved a visual representation of an action. It was shown that the integration of proprioceptive information by involving different hand positions is more relevant for 1PP imagery than for 3PP imagery leading to the conclusion that 1 PP is more embodied which means that it evokes motor simulation to a higher extent than 3PP imagery (Gallese, 2003, 2005). This is in line with the work of Ionta et al. (2007) who provided behavioral evidence that egocentric transformations involve the use of a motor strategy. This conclusion is based on the finding of decreased performance for biomechanically difficult unusual hands posture (hands with intertwined fingers kept behind the back vs. usual posture implemented by hands on the knees). The embodied nature of 1PP MRs is also supported by neuroanatomical evidence (Ionta et al., 2011a,b). The studies of the work group of Ionta revealed that the temporo-parietal junction (TPJ) plays an important role in multi-sensory integration of body-related information such as 1PP. Impairments of the TPJ are associated with decreased performance in self-other tasks (Samson et al., 2005). Interestingly, according to neuroimaging studies, egocentric transformations primarily activate the posterior parietal cortex, the frontal cortex, and the TPJ (Zacks et al., 1999; Thakkar et al., 2009). Based on these findings, we assumed that egocentric transformations are more embodied than object-based ones because of a higher activation of the motor system being the neural substrate of the body-based simulation process. The conclusion that 1PP imagery can be equated with egocentric transformation is supported by cognitive neuroscience literature: According to Lorey et al. (2009), 1PP imagery evoked a stronger activation in motor and motor-related structures of the left hemisphere compared to the 3PP condition. Whereas object-based transformations seem to be associated with right hemisphere activation, egocentric transformations primarily activate areas in the left hemisphere (Thakkar et al., 2009).

What does this embodiment approach mean with regard to the influence of fear and anxiety on MR performance? Next to the postulation of “cognitions” being embodied, there is also a strict coupling between emotion and sensory-motor integration (Gallese, 2005). For example, Adolphs et al. (1999) revealed that patients with damaged sensory-motor cortices showed decreased performance in rating or naming facial expressions. Furthermore, Wicker et al. (2003) found a common neural basis for seeing and feeling the emotion of disgust. Hence, it is concluded that perceiving an emotional stimulus and experiencing an emotion both might involve highly overlapping mental processes. Gallese (2003) has recently applied the idea that our empathic responses to everyday images might depend on the activation of mirror-neuron mechanisms. A mirror neuron is a neuron that is supposed to fire both during the execution and the observation of a given behavior (Gallese and Sinigaglia, 2011). Niedenthal (2007) tentatively proposes, that this also holds true for emotions. Hence, mirror neurons might appear to “imitate” the behavior and emotion of another person by a kind of motor simulation. Motor simulation in turn is the crucial feature of egocentric transformations. Even though it is quite speculative at this point, we assume that the influence of fear is more pronounced for egocentric than for object-based transformation because of the higher motor simulation being the essential link between embodied emotions and cognitions. Since this is a simulation-based account, this assumption is restricted to the influence of fearful stimuli and does not involve the factor “anxiety” as personality trait.

### GOAL OF THE STUDY

The present investigation differs from previous research by investigating the influence of fear and anxiety on MR performance with focus on the differentiation of egocentric and object-based transformations. Based on the work of Borst et al. (2012), we created an emotional version of the MR test by presenting fearful vs. neutral stimuli previous to the each MR trial. Concerning stimulus material, Borst et al. (2012) used Shepard–Metzler three-dimensional objects. In our study, we had two object-based conditions with pairs of letters and human figures and one egocentric MR task where one single human figure was presented. In contrast to their work, we did not use a Median split to define two groups with higher and lower scores on the scale of the state-trait anxiety test (STAI). This inventory measures two types of anxiety: “trait anxiety” which is anxiety as personality trait and “state anxiety” considered to be an anxiety related to a specific situation (Spielberger et al., 1983).

According to MacCallum et al. (2002), many problems occur when a continuous variable is turned into a categorical one: (1) median splits alter the original information. After dichotomization, persons within one group may differ more in their scores than persons in different groups. (2) Effect sizes get smaller both in correlations, ANOVA, and regression which represents a loss of statistical power. (3) Concerning the analyses with two independent variables, the chance of finding spurious statistical significance and the overestimation of effect size is increased. (4) Measurement reliability is reduced. The dichotomization of anxiety is justified by MacCallum et al. (2002) only in rare situations like having clear distinct categories based on the diagnosis by a therapist, for example. In line with these negative consequences, we preferred to include “trait anxiety” as co-variate. We restricted the analysis to trait-scores instead of state-scores because we focus on the influence of anxiety as personality trait on MR performance.

Concerning the role of fear, in line with the notion of an enhanced activation of the amygdala that in turn modulates activity in parietal areas through the presentation of aversive stimuli we expected that fear primes MR performance. It is still an open question if anxiety affects MR performance and if so, to what extent, but based on the negative influence of anxiety on cognitive processes (cf. Paulman and Kennelly, 1984; Tohill and Holyoak, 2000) it is reasonable to conclude that anxiety has a negative influence on MR performance as well. Additionally, we wanted to investigate if the influence of fear on MR performance is the same for egocentric and object-based transformations. Based on the embodied cognition viewpoint which argues for a common basis of emotions, cognitions, and motor-related structures, we assumed a stronger link between egocentric transformations and fear compared to object-based ones because of the higher motor simulation in perspective transformations.

## MATERIALS AND METHODS

### PARTICIPANTS

Eighty-six adults, 43 men (mean age: 23.27, SD = 4.27) and 43 women (mean age: 21.36, SD = 1.72) participated and were classified into the following two types of emotion: negative and neutral. The “negative emotion” group consisted of 22 men and 21 women, the “neutral emotion” group was composed of 21 men and 22 women with no significant difference in both age (mean age_negative_: 23.27, SD = 4.27; mean age_neutral_: 23.27, SD = 4.27), *t*(84) = -0.69, *n.s.,* and the anxiety trait score (mean STAI_negative_: 35.86, SD = 8.72; mean STAI_neutral_: 36.67, SD = 7.62), *t*(84) = -0.46, *n.s.* Regarding intelligence, they showed comparable scores (mean IQ_negative_: 114.74, SD = 13.36; mean IQ_neutral_: 115.49, SD = 13.20), *t*(84) = -0.26, *n.s.*, see **Table 1**. Participants were recruited through advertisement at the university. All participants received either €10 for participation or credits for psychology courses. None of the participants have participated before on MR tests. All participants gave informed consent for participation.

**Table 1:** Population description (mean and SD).

Emotion	Negative mean (SD)	Neutral mean (SD)	*T*	*p*-Value
Age	22.56 (4.32)	22.05 (2.18)	0.693	0.490
IQ	114.74 (13.35)	115.49 (13.20)	0.231	0.796
STAI	35.86 (8.72)	36.67 (7.62)	-0.461	0.646

### APPARATUS AND STIMULI

#### Cognitive speed (ZVT; Oswald and Roth, 1987)

Cognitive speed was measured with the Number Connection Test (Zahlenverbindungstest; ZVT; Oswald and Roth, 1987). In total, the test administration, including instructions and practice matrices, takes about 10 min and consists of four sheets of paper. On each sheet, the numbers 1–90 are presented in a scrambled order in a matrix of 9 rows and 10 columns. The participants had to connect the numbers as fast as possible in ascending order, and the correct connected numbers were analyzed. From the obtained ZVT-scores, IQ values could be estimated. The correlation ranged between *r* = 0.60 and 0.80 (Vernon, 1993). The internal consistency as well as 6-month test–retest reliability of the ZVT is about 0.90–0.95. The test administration, including instructions and practice matrices, takes about 20 min.

#### State-trait anxiety test (Spielberger et al., 1983); German Version (Laux et al., 1981)

The state-trait anxiety test measures trait and state anxiety, with 20 questions concerning state and 20 questions concerning trait anxiety. Internal consistency is about 0.86 to 0.95; 2-month test–retest reliability coefficients is about 0.65–0.75 (Spielberger et al., 1983).

#### Mental rotation test

**FIGURE 1:**
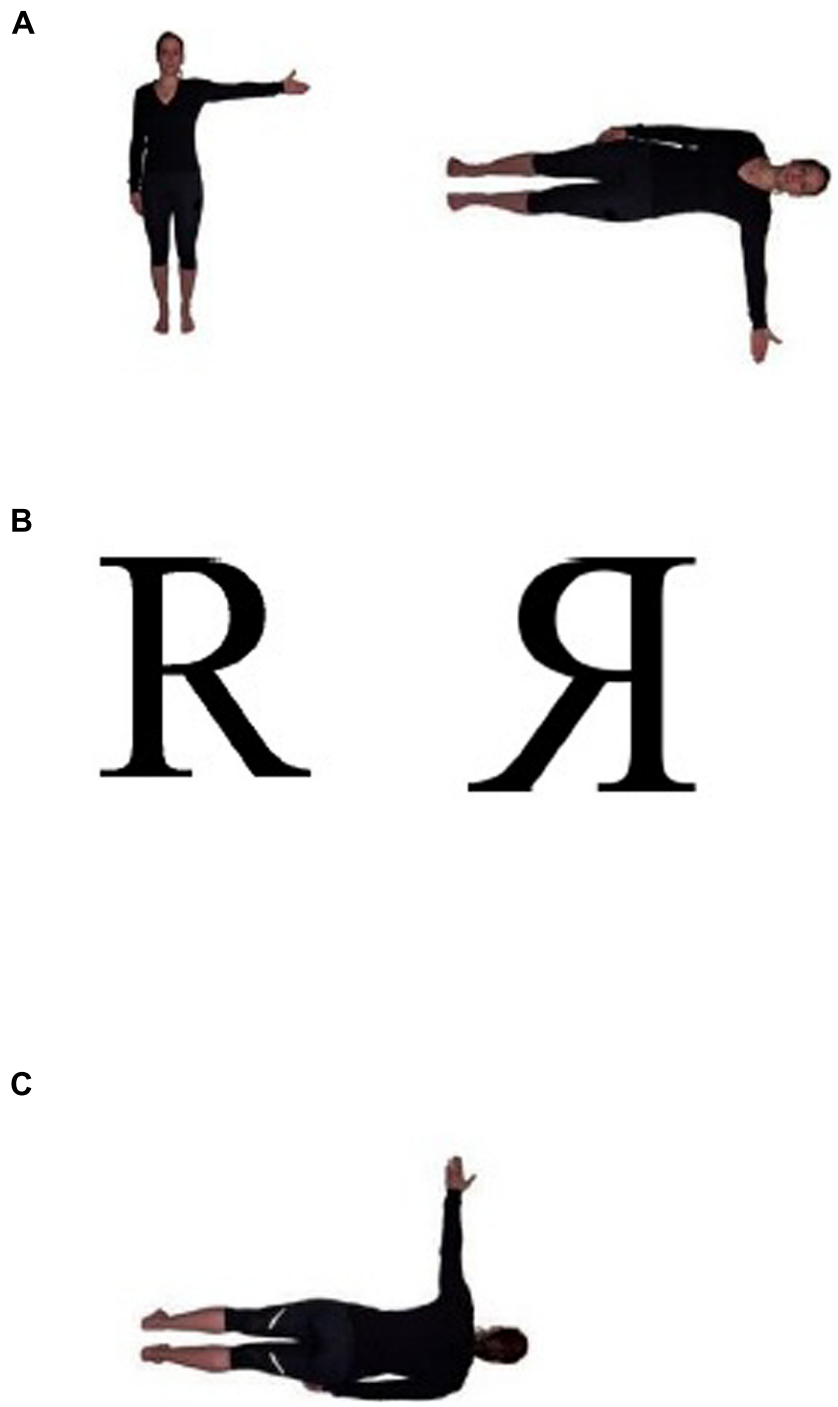
**Examples of the three different conditions: (A) body figures object based (BFO), (B) letters, and (C) body figures egocentric, (BFE)**.

The MR task was run on a laptop with a 17″ monitor located approximately 60 cm in front of the participant. The stimuli types were adapted from the work of Steggemann et al. (2011) and already used in a study with older participants with different angular disparities (Jansen and Kaltner, 2014). They consisted of three experimental types, (a) frontal view of two female people with either the left or the right arm extended (body figure object based: BFO), (b) front and back view of one female person with either the left or right arm extended (body figure egocentric: BFE), and (c) the letters R and F, see **Figure 1**. The letters were black and the human figures were wearing black clothes.

In the letter and BFO conditions two drawings of the same kind of stimuli were presented simultaneously with an angular disparity of 0, 30, 60, 90, 120, 150, or 180°. The right stimulus was obtained by the rotation of left stimulus, the so-called comparison figure. Half of the trials were pairs of identical objects and half were mirror-reversed images. We decided to use two object-based conditions to control whether negative emotions could affect MR performance of different types of stimuli. This assumption is based on the work of Amorim et al. (2006), who provided body characteristics to three-dimensional Shephard–Metzler (S-M) cubes to suggest a human posture to trigger a body analogy process in a same–different judgment task. They showed that adding body characteristics to S-M cubes increased performance compared to the S-M cubes without these characteristics because this spatial embodiment improved object shape matching. In the BFE condition, only one figure raising the left or right arm was presented in the rotation angle mentioned above. All stimuli were rotated in the picture plane.

**FIGURE 2:**
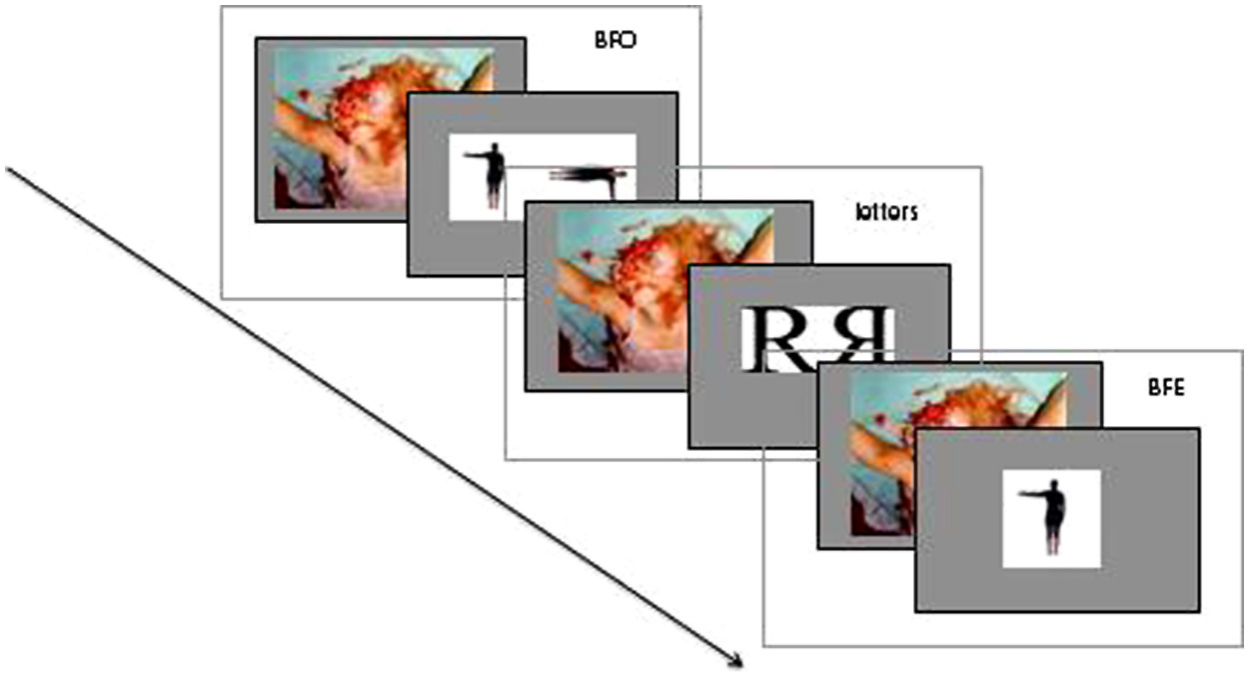
**Procedure including blocks of three conditions (BFO, letters, BFE) with a negative picture (negative emotion group) before each trial**.

Before each trial pictures from the International Affective Picture System (IAPS) were presented, as illustrated in **Figure 2**. This picture gallery includes a large set of standardized, emotionally evocative, colored photographs that represent three categories of affective stimuli: negative, neutral, and positive ones. We concentrated on the comparison between negative and neutral images. Therefore, the valence of the pictures (negative, neutral) served as between-subject factor. Since the IAPS consisted of 193 negative and 130 neutral pictures, which is not sufficient for the 336 MR trials in total, we had to choose 112 images for each block resulting in three repetitions of each image. Even if habituation and consequently a decrease of emotional response were risked, a less amount of trials would not have been arguable from the scientific viewpoint. The selection of the images was randomized. However, this selection was based on the fact that level of arousal and valence was comparable for both emotions. For this purpose, the IAPS provides a list with scores of valence and arousal. The primes were controlled for both levels. According to Borst et al. (2012), the presentation lasted for 75 ms.

### PROCEDURE

The individual test sessions lasted about 60 min and took place at a silent room at the University. At the beginning the participant completed the demographic questionnaire, the state-trait anxiety inventory and the ZVT.

Later, the MR test with standardized task instruction was conducted. In the BFO and letter conditions, participants had to press the left mouse button (left-click) when the two stimuli were “same” and the right mouse button (right-click) when the two stimuli were “different.” In this case “same” means that the stimulus on the right side was identical to the comparison stimulus, “different” means that the stimulus on the right side was not identical to the comparison stimulus. In the BFE condition participants had to decide if the figure had the right (right mouse click) or the left arm (left mouse click) outstretched (see Jansen and Kaltner, 2014).

According to Jansen and Kaltner (2014), three blocks with 112 trials of one transformation condition were presented in randomized order. After every ten trials within each block, a pause of 15 s was given before the next ten trials were administered. There were eight practice trials before each block. Each trial began with a fixation cross for 1 s. After that, the pair of stimuli appeared and stayed on the screen until participants answered. Feedback was given for 500 ms after each trial: for correct responses a “+” appeared in the center of the screen and for incorrect responses a “–” appeared. The next trial began after 1500 ms.

Each participant performed three blocks of 112 experimental trials, resulting in 336 trials: 3 transformation types (BFE vs. BFO vs. letters) * 2 trial types (same vs. different/left vs. right) * 7 angular disparities (0°, 30°, 60°, 90°, 120°, or 150°) * 4 repetitions of each combination * 2 stimuli per block (BFO: left vs. right; letters: R, F; BFE: front vs. back). In each block, the order of the presentation of the stimuli was randomized.

### STATISTICAL ANALYSIS

First, to exclude that the MR performance is influenced by possible IQ differences between gender and group an univariate analysis with the dependent measure IQ and the independent variables gender and group was conducted.

Second, two repeated analyses of variance were conducted, with “reaction time” and “accuracy rate” as dependent variables, and with “angular disparity” (0°, 30°, 60°, 90°, 120°, 150°, 180°), “transformation type” (BFO, letters, BFE), “emotion” (negative, neutral) as factors. The factors “angular disparity” and “transformation type” were the within-subject factors. Because preliminary analysis revealed no relevant effect with the factor gender only the factor “emotion” (negative vs. neutral pictures) served as between-subject factors. The variable “trait anxiety” was included as a co-variate. For reaction time, only the responses for “same” trials were analyzed because angular disparity is not clearly defined for mirror-reversed responses (Jolicśur et al., 1985). For error rates, the PR score and the accuracy rate as well was calculated. The PR score (which is the abbreviation for the discrimination index according to Snodgrass and Corwin, 1988) was calculated for each angular disparity (30°, 60°, 90°, 120°, 150°, 180°). It is defined as the difference between the hits % (% of “same” responses for trials where “same” was the correct response) and the false alarm % (% of “same” responses for trials where “same” was the incorrect response). This specific bias measure is based on the two-high threshold (2HT) model of recognition (Snodgrass and Corwin, 1988) and is used in recognition (Tulving and Thomson, 1971) and decision tasks (Schoppek, 2001). A high PR score is associated with good discrimination performance, whereas low scores argue for random performance (Schoppek, 2001). This specific index is suggested by Woodworth (1938) for reasons of correction of guessing. It is considered to prohibit guessing by always pressing the same button and trough chance hits because it is based on the 2 HT-model where error-variance specific to guess-responses is kept minimal trough providing sensitive ( = “high”) thresholds (Coombs et al., 1970; see Jansen et al., 2013). The additional analysis of the accuracy rate was conducted for a better understanding whether the tasks were comparable by seeing the raw accuracies.

Third, a repeated analysis of variance was calculated with “MR speed” as a dependent variable and “transformation type” as within-subject factor and “emotion” as between-subject factor. The variable “trait anxiety” was included as a co-variate. MR speed was calculated as the inverse of the slope of the regression line, calculated separately for each subject, relating RT to angular disparity and was expressed as degrees per second. A higher MR speed means that a larger angular disparity is rotated per second. According to the traditional theory of MR (Heil and Rolke, 2002) claiming several stages of MR, MR speed is interpreted as the MR process itself, whereas overall reaction times include stages such as perceptual preprocessing, identification of the stimulus and its orientation, judgment of the parity, and response selection (Heil and Rolke, 2002).

The significance levels of the analyses of variance results were corrected according to the method of Greenhouse–Geisser to compensate for non-sphericity of the data if necessary.

## RESULTS

### ZVT

There were neither gender differences, *F*(1,84) = 0.38, *n.s.* nor a group effect, *F*(1,84) = 0.008, *n.s.* nor an interaction between both factors, *F*(1,84) = 1.52, *n.s.* concerning the transformed IQ values.

### MENTAL ROTATION

#### Reaction time

Concerning reaction time, the analysis of variance showed three main effects for the factors “transformation type,” *F*(1,85) = 10.13, *p* < 0.001, partial *η*^2^ = 0.11, and “angular disparity,” *F*(1,85) = 54.68, *p* < 0.001, partial *η*^2^ = 0.39. The covariate also reached significance and could be expressed by a significant correlation between “trait anxiety” and the averaged reaction time for each transformation type (BFO, letters, BFE). There were two positive significant correlations: (1) between “trait anxiety” and “BFO” (*r* = 0.26, *p* < 0.05), 2) between “trait anxiety” and “letters” (r = 0.23, *p* < 0.05). Regarding the significance of the factor “transformation type,” Bonferroni-corrected *t*-tests showed that the reaction was higher for the BFO condition (*M* = 996.70, SD = 21.55) compared to the letter condition, (*M* = 747.29, SD = 15.57), *t*(1,85) = 12.84, *p* < 0.001, and to the BFE condition, (*M* = 924.70, SD = 15.96), *t*(1,85) = -12.61, *p* < 0.0001. Furthermore, there was a significant difference between the reaction time in the letters and BFE condition, *t*(1,85) = 3.21, *p* < 0.01. Regarding the main effect of the factor “angular disparity,” *post hoc* pair-wise comparisons showed higher reaction times for each consecutive angular disparity (*p* = < 0.001) except the one at 30° which did not differ from that of 0°, *t*(85) = -2.34, *n.s.*

**FIGURE 3:**
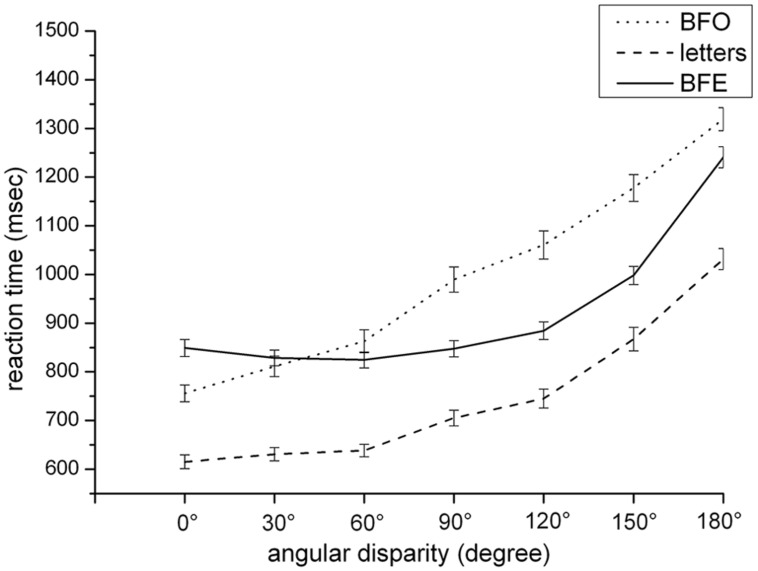
**Reaction time dependent on transformation type and angular disparity**.

Furthermore, there were two interactions:

(1) The “transformation type” × “angular disparity” interaction was significant, *F*(1,85) = 1.84, *p* < 0.05, partial *η*^2^ = 0.02, and it is illustrated in **Figure 3**. Whereas reaction time in the BFO condition was overall increasing with angular disparity and higher for each consecutive angle (*p* = < 0.001), reaction times in the letters condition did not differ between angular disparities of 0 and 30°, *t*(85) = -2.06, *n.s.,* and between angular disparities of 30 and 60°, *t*(85) = -1.01, *n.s.*. Increasing disparity in the BFE task only led to higher response times for disparities larger than 90° (*p* = < 0.001). All other effects did not reach significance at the 0.05 level. Furthermore, by trend reaction time in the egocentric transformation condition surprisingly decreased between the angular disparity of 0 and 60°. That is, whereas reaction times in the object-based condition roughly increased linearly with increasing disparity as expected, they showed a U-shaped pattern for the egocentric transformation condition.
(2) The interaction between the covariate “trait anxiety” and “angular disparity” was significant, expressed by a correlation between “trait anxiety” and the averaged reaction time for each angular disparity. The correlation between “trait anxiety” and the angular disparities of 0, 120, 150, and 180° reached significance (0°: *r* = -0.21, *p* < 0.01; 120°: *r* = -0.29, *p* < 0.01; 150°: *r* = -0.28, *p* < 0.01; 180°: *r* = -0.27, *p* < 0.01). All other effects failed to reach significance.

#### Accuracy rate

The analysis of the PR score showed one main effect of the factor “angular disparity” *F*(1,85) = 8.932, *p* < 0.001, partial *η*^2^ = 0.09. Bonferroni-corrected *t*-tests revealed that from an angular disparity of 90°, there was a lower PR score for the following angular disparity compared to the preceding one (all *p* < 0.001). All other effects did not reach significance.

Regarding accuracy rate, results revealed two significant main effects of the factors “transformation type,” *F*(1,85) = 5.11, *p* < 0.01, and “angular disparity,” *F*(1,85) = 136.28, *p* < 0.001. According to multiple *post-hoc* comparisons, participants showed higher accuracy rates in the BFE and the letters condition compared to that found in the BFO condition. Performance between the letters and BFE condition did not differ. Regarding the main effect of the factor “angular disparity,” the decrease of accuracy emerges for disparities larger than 90° (*p* = < 0.001). All other effects did not reach the 0.05 significance level.

**FIGURE 4:**
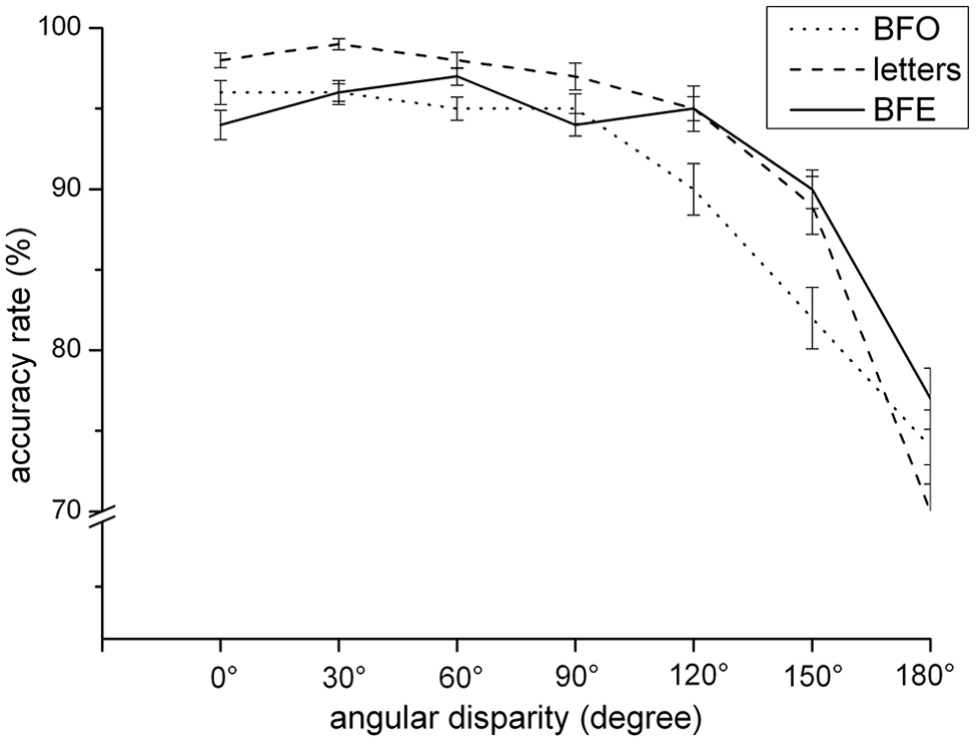
**Accuracy rate dependent on transformation type and angular disparity**.

Furthermore, the interaction between “transformation type” and “angular disparity” was significant, *F*(1,85) = 5.00, *p* < 0.001. Whereas accuracy rate in both object-based conditions (BFO, letters) was overall decreasing with angular disparity by trend and significant lesser for each consecutive angle from an angular disparity of 90° on (*p* = < 0.001), increasing disparity in the egocentric task only led to higher error rates for disparities larger than 120° (*p* < 0.001). Furthermore, by trend accuracy rate in the egocentric transformation condition surprisingly increased between the angular disparity of 0 and 60°, and between 90 and 120°. All other effects did not reach significance at the 0.05 level. Together, this small-angle advantage is more pronounced for the object-based transformations than for the BFE condition, as illustrated in **Figure 4**.

#### Mental rotation speed

Due to negative rotation speed, two persons had to be excluded. The analysis of variance showed a significant main effect of “transformation type,” *F*(1,85) = 3.91, *p* < 0.05, partial *η*^2^ = 0.05. Bonferroni-corrected *t*-tests showed that participants rotated stimuli in the BFE condition (*M* = 693.77°/s, SD = 61.99) significantly faster than those in the BFO condition (*M* = 348.92°/s, SD = 11.65), *t*(1,85) = -5.67, *p* < 0.001, but not significantly faster than letters (*M* = 581.57°/s, SD = 43.97), *t*(1,85) = -1.44, *n.s.*

**FIGURE 5:**
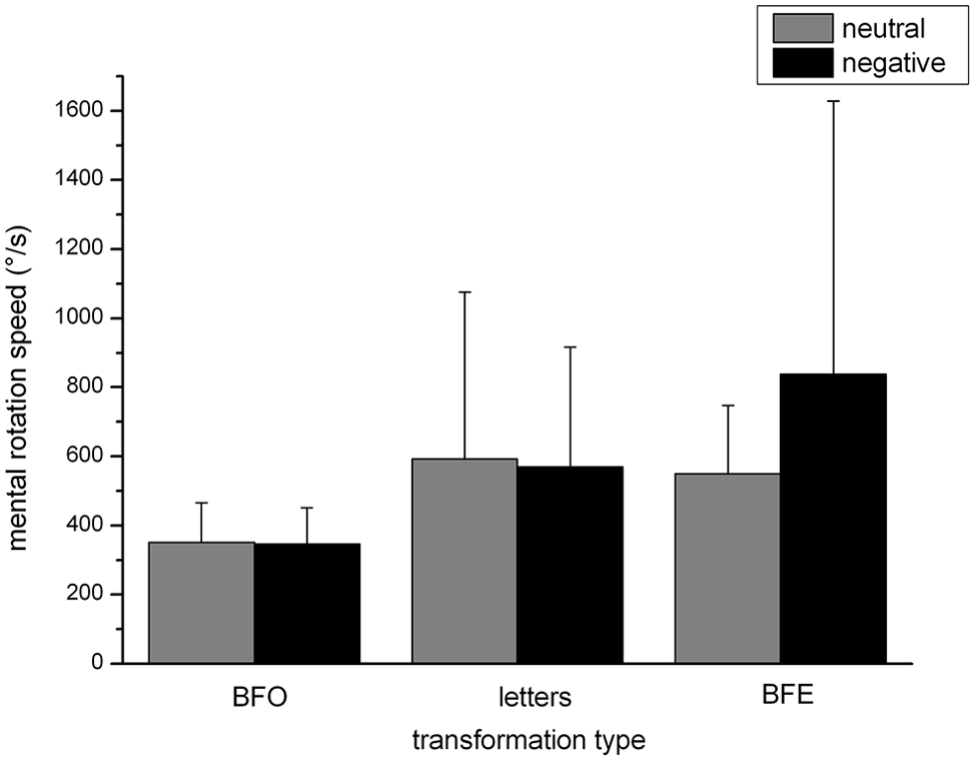
**Mental rotation speed dependent on transformation type and emotion**.

Furthermore, results showed two significant two-way interactions:

(1) The interaction between “transformation type” and “emotion” reached significance at the 0.05 level, *F*(1,85) = 5.37, *p* < 0.001, partial *η*^2^ = 0.07. *Post-hoc* comparisons showed that the rotation speed did not differ for participants who have either seen negative or neutral pictures before the MR task, both in the BFO condition, *t*(1,84) = -0.18 *n.s.*, and in the letters condition, *t*(1,84) = -0.26 *n.s.*, whereas MR speed differed between both groups in the BFE condition, *t*(1,84) = 2.32, *p* < 0.05. The MR speed for the BFE condition was much higher if participants had seen negative pictures (*M* = 838.08, SD = 791.18) compared to neutral ones (*M* = 549.46, SD = 198.02), see **Figure 5**. All other effects were not significant.
(2) The interaction between “transformation type” and the co-variate “trait anxiety” was significant, *F*(1,85) = 4.61, *p* < 0.05, partial *η*^2^ = 0.05 expressed by a negative correlation between “trait anxiety” and the rotation speed of “letters” (*r* = -0.26, *p* < 0.05).

## DISCUSSION

The main goal of our study was to investigate if the influence of fear on MR performance is the same for egocentric and object-based transformations. Furthermore, we wanted to examine the effect of anxiety on MR performance. The main results were a facilitation effect of fear which is restricted to egocentric transformations: participants rotated stimuli in the BFE condition more quickly after seeing an aversive image compared to a neutral one. Concerning the influence of anxiety, individuals with high scores on the trait-anxiety scale of the STAI showed both partially higher reaction times and a partially slower MR speed.

### EFFECTS OF FEAR ON MENTAL ROTATION PERFORMANCE

In line with previous findings (Borst et al., 2012; Borst, 2013), we could replicate an influence of fear on MR performance. Our results confirm the enhanced effect expressed in a higher MR speed after the presentation of fearful images compared to neutral stimuli. However, according to our results, this effect is transformation specific: it is restricted to egocentric rotations. Therefore, fear seems to influence egocentric transformations to a higher extent than object-based ones.

Like mentioned above, both types of strategies differ in a crucial point: Whereas in object-based transformations participants are asked to mentally move/rotate the object in relation to the surrounding environment, in an egocentric chronometric MR tasks people are required to change their own perspective because they have to imagine themselves rotating in order to complete the task (Zacks et al., 2002; Devlin and Wilson, 2010; Kessler and Rutherford, 2010). Therefore, we tentatively propose that in egocentric transformations there is a stronger link between the (bodily) self and the type of task compared to that emerging in object-based transformations. This in turn might lead to the conclusion that the induction of an emotion like fear stronger affects transformations where the own body is required compared to rotations where the participant’s position remains fixed and MR is analogous to a manual rotation (Shepard and Metzler, 1971). This notion is supported by neuroimaging findings of Wraga et al. (2005) who showed different underlying neural structures for object-based vs. perspective transformations: whereas in object rotation, activity in pre- and primary motor areas was found which are responsible for motor representations that reflect manipulation, egocentric transformations activate areas that are involved in actual bodily movements (Zacks and Michelon, 2005).

Another possible explanation for the enhanced effect of fearful stimuli on egocentric transformation is based on the idea that fearful primes may prepare the body to react. We tentatively propose that this motor pre-activation has a stronger impact on the BFE condition which is suggested to be embodied to a higher extent than the object-based conditions (letters, BFO). The notion, that the processing of emotional stimuli activates bodily reactions has already been pointed out by Darwin (1955), in the sense of “fight or flight.” Interestingly, Oosterwijk et al. (2010) revealed, that even fear knowledge in the sense of no subjective fear experience elicits embodied reactions. Fear concept activation was induced by the following task: Participants had to unscramble neutral or fear sentences followed by the presentation of fear images. Fear activation led to increased electrodermal activity while viewing fearful pictures compared to the neutral condition. Furthermore, next to fear-induced changes in the peripheral nervous system, Ehrsson et al. (2007) revealed that premotor areas are activated under threat. Regarding the involvement of motor processes on object-based and egocentric transformations, there is plentiful evidence arguing for egocentric rotations to be more embodied compared to object-based ones. For example, Kessler and Thomson (2010) demonstrated a robust effect of the congruence between body posture and direction of egocentric rotation that is, participants responded faster when their body posture was matching with the implied rotation direction. This finding has led to the conclusion that mental object rotation is either not embodied or very differently embodied because in this condition this congruence effect was less pronounced. However, this kind of body feedback is focused on static movement. Given the situation, that the body is prepared to react in the sense of “approach or avoidance” after having seen a fearful stimulus, further embodiment research should be taken into account which concentrates on changes in the form or the direction of the movement. This was the main goal of Cacioppo et al. (1993) who created an avoidance condition where participants had to put pressure on a table away from the own body vs. an approach condition that induced pressure toward the body from below a table. The attitude toward Chinese ideographs being rated as “neutral” before served as dependent variable. The results showed that the approach movement produced more positive attitudes compared to the avoidance condition. However, applied to the present study, even if no real movement takes place, but rather a pre-activation which means that motor simulation is primed in a certain manner, we nevertheless tend to conclude that motor pre-activation through fearful stimuli has a stronger impact on the BFE condition where a higher involvement of motor simulation is supposed. The influence of this specific kind of motor-priming against the background of the embodied cognition approach represents an interesting topic for future research and should deserve enhanced attention.

Another attempt to explain the egocentric-specific influence of fear could involve the meaning of the working memory (WM). We tentatively propose that fear impairs functions of the WM which affects object-based rotations to a higher extent. This assumption stems from the fact that in egocentric transformations the visual buffer being the neuronal substrate for both imaginal and perceptual visuo-spatial transformations is not that highly loaded because there is no image interference in left–right judgments tasks (Zacks et al., 2002). The widespread definition of WM according to Baddeley and Hitch (1974) refers to the ability to maintain task-relevant information in a system while simultaneously performing a cognitive task. The involvement of the WM in MR performance relies on the following process: subsequent to the actual MR the imagined stimulus must be aligned with the comparison stimulus. Therefore, the information of this specific sub-process must be maintained to enable access to information during the next stage. The involvement of the visuo-spatial sketchpad, a subsystem of the WM, in MR is provided by Lehmann et al. (2014). The researcher revealed a positive correlation between spatial-WM capacity measured by the Corsi block tapping task and MR performance. Interestingly, whereas no variance was explained by motor performance, 55.5% of the variance was explained by the predictor’s digit span forward and Corsi forward according to their results. The notion, that processes of encoding and comparing represent functions of the WM, is supported by the work of Booth et al. (2000) who demonstrated that mental rotated stimuli are temporally stored in WM. We tentatively propose that the presentation of fearful stimuli distracts awareness and therefore capacity available for processing. In support of this notion, there is empirical evidence that increased emotionality, and especially stress, impairs WM (Diamond and Park, 2000; Kim and Diamond, 2002). Applied to object-based and egocentric transformations of the present study, object-based rotations seem to be affected to a higher extent because WM is assumed to be involved stronger compared to perspective transformations, as mentioned above. In line with this MR speed should be specifically slowed in the object-based conditions (BFO, letters). This idea is supported by our results. Higher MR speed restricted to the egocentric condition could therefore be interpreted as advantage due to a lesser WM influence in this type of transformation. Although very speculative, the meaning of the WM in MR processes and its functions under fear should be investigated in more detail.

A further approach for this finding could be attributed to a conceptual link between visuo-spatial perspective taking and perspective-taking in the abstract sense, specifically empathy. Although purely speculative, it could be concluded that the presentation of aversive stimuli elicits a stronger activation of areas which are the neural correlate for egocentric transformations and therefore represent the social construct empathy in an abstract sense compared to those being activated during object-based transformations. In perspective transformations, subjects are required to transform themselves into the body of another person. There is evidence that this kind of self-other equivalence is a basic condition for empathy (Gallese, 2003). Allport’s (1937, p. 530) definition of empathy like “putting oneself in the place of another” underlines the proposed link between these two components and is theoretically supported by the framework of embodied cognition, mentioned in the section “Introduction.”

Based on the finding of the activation of the parietal cortex during both visuo-spatial processes and empathy (Preston and de Waal, 2002), Thakkar et al. (2009) investigated this relationship by exploring the correlation between a self-other transformation task and self-reported empathic concern. They used a task of spatial attention as well to assess the hemispheric dominance. They found positive correlations between rightward biases and self-reported empathy which suggests a left hemisphere lateralization of this personality trait. Since egocentric transformations lead to increased activation of the left hemisphere as well, this parallelism of lateralization of egocentric transformations and empathy seems to support our notion of the link between these two components. By using the interpersonal reactivity index, four subscales of self-reported empathy were assessed: Perspective-Taking (PT), Fantasy (FS), Empathic Concern (EC), and Personal Distress (PD). The PT subscale measures the ability to adopt the psychological viewpoint of others and the FS scale assesses the tendency to put oneself in the feelings of fictitious characters. Empathic concern corresponds to “other-oriented” feelings of sympathy and concern for others in unfortunate situations. Personal Distress assesses “self-oriented” feelings of personal anxiety and the discomfort in tense interpersonal situations (Davis, 1980). Furthermore, it is associated with susceptibility to emotion contagion (Doherty, 1997).

In contrast to their expectation, Thakkar et al. (2009) found that speed of visuo-spatial self-other transformations correlated with decreased empathic concern in women. It was assumed that the good performance derived from the high level of testosterone in the female participants which is related to both better spatial abilities (Broverman et al., 1981) and decreased empathy (Chapman et al., 2006). However, women with increased scores of PD showed faster self-other transformations. Less time needed for this kind of transformation was attributed to a less distinct representation of self and other which is reflected in a high tendency to emotion contagion where the affective state of another person is adopted in a way that it cannot be differentiated from the own feeling anymore (Thakkar et al., 2009). This leads to the assumption that the supposed relationship between self-other transformation in MR and empathy has to be interpreted with respect to specific subscales. It could be an interesting issue for future research to combine the design of Thakkar et al. (2009) with our study by investigating the relationship between empathy and visuo-spatial transformations with regard to the influence of fear on MR performance.

### EFFECTS OF ANXIETY ON MENTAL ROTATION PERFORMANCE

The positive correlation between “trait anxiety” and “reaction times” of letter and BFO condition and “MR speed” of letters in our study suggests that higher scores in the trait anxiety scale of the STAI are associated with higher reaction times of these conditions. These results are contradictory to the finding of previous research showing a facilitation effect of emotion: participants with high state-anxiety rotated objects more quickly after the presentation of fearful faces compared to neutral ones (Borst et al., 2012). However, there are two reasons that complicate a direct comparison: (1) Whereas Borst et al. (2012) used the state-anxiety scale, we decided to assess the trait-anxiety scale because we wanted to emphasize the influence of anxiety as personality trait on MR performance. (2) We did not use the Median split to contrast high- vs. low–anxiety group because of statistical limitations. Therefore, analyses with “anxiety” as factor were not conducted.

The findings of our study show that high anxiety scores interfere with MR performance which is in line with the negative influence of anxiety found in previous literature (cf. Paulman and Kennelly, 1984; Tohill and Holyoak, 2000). According to Bishop et al. (2004), anxiety is associated with increased distractibility, poor concentration and heightened responsivity to threat. Furthermore, anxious individuals show less attentional control over threat-related stimuli which results in a strong allocation of attention. This increased attentional capture by threat-related stimuli is attributed to the hyper-responsive pre-attentive threat-detection system centered on the amygdala (Mathews et al., 1997). In more recent research, this assumption has been modified by integrating the influence of prefrontal cortical mechanisms (Bishop et al., 2004). Bishop (2009) revealed that trait anxiety is associated with reduced recruitment of prefrontal attentional control even in the absence of threat-related stimuli which were avoided in this task.

According to Karadi et al. (2001) focused attention is one of several sub-processes playing an important role in MR performance. The traditional theory of MR differentiates five independent information-processing stages of MR (Shepard and Cooper, 1982). These are: (1) perceptual preprocessing, (2) identification/discrimination of the character and identification of its orientation, (3) MR, (4) judgment of the parity, and (5) response selection and execution (Heil and Rolke, 2002). MR itself requires the participant to imagine rotating letters to the upright position (Cooper and Shepard, 1973). This stage involves active manipulation of visual representation which is presumably more a controlled process of voluntary attention than an automatic one. This may lead to the conclusion that reduced attentional control in participants with high anxiety scores may explain their impaired MR performance. However, it still remains unclear if this kind of attention-deficit in high-anxious individuals plays a role in the attentional process involved in MR.

Interestingly, with respect to the simulation-based account the influence of anxiety is restricted to the two object-based conditions (letters, BFO) where no motor simulation was required. This is in line with the specific effect of fearful stimuli being restricted to the egocentric transformation. Both results could provide further evidence for the importance of motor simulation in the assumed link between embodied cognitions and emotions.

### LIMITATIONS

The investigation of the influence of fear by presenting aversive images is widespread. However, it still remains unclear to which extent this type of stimulus material elicits emotion. Furthermore, it raises the question which kind of emotion is triggered, whether it is rather disgust than fear. Even if the images were standardized regarding valence and arousal, the extent of the emotional response stays individually. This could be controlled by measuring the physiological response, specifically skin conductance response. However, this measurement still makes no statement about the quality of emotion. Conducting self-reported measurements could clarify the emotional state, but they can only be assessed after the MR task which may be a too long period after the presentation of the aversive stimuli.

Thakkar et al. (2009) found that speed of visuo-spatial self-other transformations correlated with decreased empathic concern in women which contradicted their expectations. Gender differences were no analyzed in our study, but could be very interesting especially with regard to the effects of fearful stimuli on MR. It could be assumed that women score higher on the empathic inventory than men and therefore show increased emotional responses which lead to enhanced MR performance, specifically concerning egocentric transformations.

Furthermore, the direct comparison between egocentric and object-based transformations should be reconsidered in view of the fact that these types of transformations differ in some aspects: visual stimulation (2 stimuli vs. 1 stimulus, cf. Zacks et al., 2002), type of judgment (same–different vs. left–right, cf. Steggemann et al., 2011) and instruction (Borst et al., 2011). Regarding the latter factor, an additional control by asking the participants how much they felt able to follow the two different instructions would have given more information about the strategy they used in the end. This as well as all the other confounding factors mentioned above should be taken into account for future research.

Taken together, the explanation approaches mentioned above based on neuronal correlates still remain quite speculative at this point since no brain activity was measured in the present study. Further behavioral and neuroanatomical research is needed to clarify this specific link between emotion and cognition.

## CONCLUSIONS

Empathy seems to be associated with egocentric transformations based on the co-activation of parietal areas during visuo-spatial processes and this social construct (Preston and de Waal, 2002). We hypothesized that aversive stimuli would enhance reactions in participants with high scores in an empathic inventory and therefore lead to a facilitation effect of fearful stimuli on MR performance. Because of the link between empathy and egocentric transformations, stronger effects compared to object-based transformations are expected. The assessment of this social construct would clarify this assumption which was not made in our study. Since certain scales of the interpersonal reactivity index like personal distress are more associated with fearfulness than other scales (Davis, 1983), this finding must be taken into account for future interpretations. Comparing egocentric and object-based transformations in clinical samples like psychopaths who lack empathy represents an interesting focus for future research.

To complete the emotional version of the MR task, the influence of positive images could be investigated in future. If the poor performance of anxious individuals is really stemming from an attentional deficit caused by threat-related stimuli, the adding of positive stimuli may clarify this assumption.

The relationship between emotion and MR seems to be very close. The findings of our study suggest that there is a facilitation effect of fear which is restricted to egocentric transformations. To what extent empathy as social construct plays a role still remains unclear and demands a lot of future research. In contrast to Borst et al. (2012) individuals with high anxiety scores show impaired MR performance after the presentation of fearful stimuli. Further research is needed to clarify which role do attentional impairments play, and more specifically: to what extent and which kind of attention is required in the five independent information-processing stages of MR mentioned above. MR seems to be an adequate paradigm to investigate the importance of both empathy and attention in the relationship between fear, anxiety and visuo-spatial processing.

## Conflict of Interest Statement

The authors declare that the research was conducted in the absence of any commercial or financial relationships that could be construed as a potential conflict of interest.
